# The decline of 6‐thioguanine nucleotides is not linked to impaired efficacy or safety of thiopurines in pregnant women with inflammatory bowel disease

**DOI:** 10.1002/bcp.70520

**Published:** 2026-03-18

**Authors:** Dianne G. Bouwknegt, Paola Mian, Femke Groen, Denise van den Berg‐Zuiddam, Andrea E. van der Meulen, Femke Crouwel, Nanne K. de Boer, Debbie S. Deben, Dennis R. Wong, Luc J. J. Derijks, Gerard Dijkstra, Arno R. Bourgonje, C. Janneke van der Woude, Marijn C. Visschedijk

**Affiliations:** ^1^ Department of Gastroenterology and Hepatology University Medical Center Groningen, University of Groningen Groningen the Netherlands; ^2^ Department of Clinical Pharmacy and Pharmacology University Medical Center Groningen, University of Groningen Groningen the Netherlands; ^3^ Department of Gastroenterology and Hepatology Leiden University Medical Center, Leiden University Leiden the Netherlands; ^4^ Department of Gastroenterology and Hepatology Amsterdam University Medical Center, University of Amsterdam Amsterdam the Netherlands; ^5^ Amsterdam Gastroenterology Endocrinology Metabolism (AGEM) Research Institute Amsterdam University Medical Center Amsterdam the Netherlands; ^6^ Department of Clinical Pharmacy, Pharmacology & Toxicology Zuyderland Medical Center Sittard‐Geleen/Heerlen the Netherlands; ^7^ Department of Clinical Pharmacy and Pharmacology Máxima Medical Center Veldhoven The Netherlands; ^8^ Department of Clinical Pharmacy and Toxicology Maastricht University Medical Center, Maastricht University Maastricht the Netherlands; ^9^ School of Nutrition and Translational Research in Metabolism (NUTRIM) Maastricht University Maastricht the Netherlands; ^10^ The Dr. Henry D. Janowitz Division of Gastroenterology, Department of Medicine Icahn School of Medicine at Mount Sinai New York NY USA; ^11^ Department of Gastroenterology and Hepatology Radboud University Medical Center Nijmegen the Netherlands

**Keywords:** 6‐thioguanine nucleotides, inflammatory bowel disease, monitoring, pregnancy, thiopurines

## Abstract

**Background:**

Thiopurines are used to maintain remission in inflammatory bowel disease (IBD). These drugs are metabolized into 6‐thioguanine nucleotides (6‐TGN), associated with efficacy, and 6‐methylmercaptopurine ribonucleotides (6‐MMPR), associated with adverse drug reactions. Pregnancy has been linked to a shift in thiopurine metabolism, characterized by reduced 6‐TGN and increased 6‐MMPR levels. The clinical impact of these changes remains unclear. To explore the association between changes in 6‐TGN and 6‐MMPR levels with disease activity and toxicity markers in women with IBD during pregnancy.

**Methods:**

This retrospective cohort study included pregnant women with IBD who used thiopurines from six Dutch hospitals (2017–2022). Linear mixed‐effects models were used to model changes of 6‐TGN and 6‐MMPR before, during and after pregnancy, and to identify associations with markers of disease activity (faecal calprotectin) and toxicity (alanine aminotransferase [ALT], leukocytes, platelets).

**Results:**

A total of 87 women with 100 pregnancies were included (64.4% Crohn's disease, 32.2% ulcerative colitis). A significant reduction in 6‐TGN was found in the second and third trimester, but no associations with changes in faecal calprotectin were detected. There were no significant increases in 6‐MMPR. Increases in 6‐MMPR were associated with modest elevations in ALT, but none of the other toxicity markers. Transient thrombocytopenia and leukopenia occurred thrice, and elevated liver enzymes were observed in nine pregnancies.

**Conclusions:**

Although 6‐TGN levels decreased during pregnancy, these fluctuations were not associated with increased disease activity or toxicity. No significant increase in 6‐MMPR levels was observed. Proactive monitoring may therefore not be warranted in the pregnant population.

**What is already known about this subject?**

- Pregnancy has been linked to a shift in thiopurine metabolism, characterized by reduced 6‐TGN and increased 6‐MMPR levels.

**What this study adds**

- The altered metabolite levels are not associated with impaired efficacy or safety of thiopurines during pregnancy
- Proactive monitoring of metabolites may not be warranted in the pregnant population.

## 1 INTRODUCTION

Inflammatory bowel diseases (IBD), which include Crohn's disease (CD) and ulcerative colitis (UC), are chronic conditions involving inflammation of the gastrointestinal tract. The diseases are characterized by alternating periods of exacerbations and remission. Treatment is focused on inducing and maintaining remission of disease.

Thiopurines are a group of immunosuppressants consisting of azathioprine (AZA), mercaptopurine (MP) and thioguanine (TG). Azathioprine is a prodrug of MP. Through a complex mechanism, thiopurines are metabolized both enzymatically and via phosphorylation to eventually form 6‐thioguanine nucleotides (6‐TGN), the pharmacologically active metabolites.^1^ In IBD treatment, apoptosis of activated T cells by inhibition of GTPase signalling by 6‐thioguanine triphosphate (6‐TGTP), one of the 6‐TGN's, is considered to be the main mechanism of action.^2^ 6‐methylmercaptopurine ribonucleotides (6‐MMPR) are the other main metabolites of AZA and 6‐MP. High levels of 6‐TGN and 6‐MMPR are related to myelotoxicity and hepatotoxicity, respectively.^1^

As IBD is frequently diagnosed in young adults, the disease and accompanying treatment often coincide with pregnancy. Pregnancy is associated with profound physiological changes, which may lead to altered pharmacokinetics (PK) of drugs. These changes include, but are not limited to, delayed gastric emptying and increased gastric pH, increased total body water and extracellular fluid and decreased plasma albumin concentration.^3^ Most of these physiological changes peak in the third trimester of pregnancy.^4^, ^5^

In previous studies, it was found that the PK of thiopurines may change during pregnancy: while levels of the active 6‐TGN metabolites decrease, levels of 6‐MMPR increase.^6^, ^7^ Therefore, the 6‐MMPR/6‐TGN ratio will also increase during pregnancy,^8^ suggesting a shift in thiopurine metabolism. Current guidelines recommend thiopurines to be continued throughout pregnancy,^9^, ^10^ with pregnant women receiving the same dosage as non‐pregnant women. Due to the plausible shunt in thiopurine metabolism, regular dosages could theoretically result in subtherapeutic 6‐TGN levels, leading to manifestations of maternal disease and/or supratherapeutic 6‐MMPR levels, leading to toxicity. Based on the advice of Jharap et al, 6‐TGN and 6‐MMPR levels are therefore monitored during pregnancy, preferably in the third trimester.^6^ However, no specific advice on changing the dosage following metabolite monitoring has been described. Moreover, while progress has been made in understanding the PK of thiopurines during pregnancy, the clinical relevance of these alterations with respect to therapeutic efficacy and safety remains insufficiently clarified.

This study aims to explore the association between alterations in 6‐TGN and 6‐MMPR levels and markers of disease activity and toxicity in women with IBD during pregnancy.

## 2 METHODS

### 2.1 Study design

A retrospective cohort study was performed on data collected from electronic health records (EHR) of six Dutch medical centres, including Erasmus University Medical Center in Rotterdam, University Medical Center Groningen (UMCG) in Groningen, Amsterdam University Medical Center in Amsterdam, Zuyderland Medical Center in Heerlen and Sittard‐Geleen, Leiden University Medical Center in Leiden and Maxima Medical Center in Veldhoven‐Eindhoven. Laboratory values could be extracted automatically using retrieval sheets; all other outcomes were collected manually from the EHRs of included patients. This study was approved by the Institutional Review Board of the UMCG (Groningen, The Netherlands, IRB no. 2022/014), and the need for written informed consent was waived.

### 2.2 Participants

Adult women were included if they had been pregnant between 2017 and 2022, while using thiopurines (AZA, MP or TG) as part of their routine IBD‐treatment, and if at least one thiopurine metabolite blood level was assessed during pregnancy. Patients were not included if they had indicated in their patient file that their data may not be re‐used for medical research. Patients were selected by evaluating lists of all thiopurine metabolite measurements taken from adult women within the study period in each hospital. Treating physicians or a study delegate determined whether these values were taken from women with IBD, and if the measurements had taken place during pregnancy and/or the pre‐conceptional or postpartum period.

### 2.3 Outcomes

#### 2.3.1 Variables

The collected data provide information on patient characteristics (height, weight, age) and disease characteristics (phenotype, disease duration, Montreal classification) at baseline (conception). Colitis severity was determined based on the treating physician's documented assessment, which reflects routine clinical evaluation incorporating symptoms, faecal calprotectin and endoscopic findings when available. The most severe recorded colitis activity experienced by the patient up to that point was used to classify disease severity. Dosage and frequency of thiopurine administration were logged, as well as the use of any IBD co‐medication. Pregnancy outcomes were assessed, including duration and date of pregnancy, mode of delivery and birthweight of the infant.

All available 6‐TGN and 6‐MMPR levels pre‐, mid‐ and post‐pregnancy were extracted from the EHR, including the date of sampling. For AZA and MP users, the laboratory measurements included 6‐TGN and 6‐MMPR levels. In TG users, only 6‐TGN levels were measured, as TG is metabolized via direct ribosylation to 6‐TGN and does not undergo TPMT‐mediated methylation leading to 6‐MMPR formation.^11^ Co‐medication at the time of sampling was noted.

Thiopurine metabolite levels were measured as part of routine clinical care in all centres. In most cases, metabolites were measured as part of standard monitoring according to ECCO guidelines.^9^ Additional measurements were sometimes performed after dosage changes or in response to concerns about efficacy. See “Handling of missing data” for our assessment of differences in baseline characteristics between women with and without measurements at the separate time points.

The metabolites were measured in red blood cells (RBC) by a validated high‐performance liquid chromatography (HPLC) analytical method of Dervieux and Boulieu.^12^ From 2019 onward, one hospital (UMCG) utilized a liquid chromatography–tandem mass spectrometry (LC–MS/MS) method.^13^ A subsequent comparability analysis demonstrated that the results obtained with the new method were consistent with those of the previous assay. To rule out possible differences in 6‐TGN and 6‐MMPR concentrations due to inter‐laboratory variability, analytical results of the Dutch quality control program for thiopurine measurements were evaluated for the participating hospitals. Concentrations of 6‐TGN and 6‐MMPR were comparable across most laboratories. However, the laboratory at Amsterdam University Medical Center reported 6‐TGN concentrations approximately 1.2 times higher compared to the measurements by the other laboratories. To account for this discrepancy, 6‐TGN values from this hospital were adjusted using a correction factor of 0.833 prior to data analysis.

Calprotectin was used as an indicator of disease activity, and alanine transaminase (ALT), aspartate transaminase (AST), γ‐glutamyltransferase (γ‐GT), leukocytes and platelets were used as indicators of hepatotoxicity and myelotoxicity. These values were logged if they were measured within 2 weeks before or after the corresponding 6‐TGN and 6‐MMPR measurements. In case of multiple measurements in that period, the measurement taken closest to the metabolite measurement was used.

#### 2.3.2 Definitions

The pre‐pregnancy period was defined as twelve months prior to conception. To allow a more detailed evaluation of metabolite dynamics during pregnancy, each trimester was divided into two equal intervals (first and second half). A trimester spans approximately three months, during which substantial physiological and pharmacokinetic changes can occur. Although an even more detailed approach (e.g., per month or week) would have been preferred, the number of available measurements limited the feasibility of such an analysis. The trimesters were therefore defined as follows: trimester 1–1 (up to day 45), trimester 1–2 (day 46 – day 91), trimester 2–1 (day 92 – day 141), trimester 2–2 (day 142 – day 189), trimester 3–1 (day 190 – day 235), trimester 3–2 (day 236 – birth). Postpartum measurements were collected when available until six months after giving birth.

As liver enzymes tend to modestly decrease during pregnancy,^14^, ^15^ any increase above the upper limit of the standard reference range was considered relevant and logged as a liver enzyme elevation: ALT >40 U L^−1^, AST > 35 U L^−1^, or γ‐GT > 35 U L^−1^. This is the conventional unit in clinical practice; 1 U L^−1^ corresponds to 16.67 nkat L^−1^. In addition, leukopenia (<4 × 10^9^ L^−1^) and thrombocytopenia (<150 × 10^9^ L^−1^) were noted as indicators of myelotoxicity. Alkaline phosphatase (ALP) and haemoglobin (Hb) were not used for toxicity assessment due to the pronounced physiological changes they undergo during pregnancy. ALP levels rise substantially, particularly in the third trimester,^14^ due to placental isoenzyme production, while Hb concentrations decrease as a result of haemodilution.^15^ These predictable, pregnancy‐related shifts in these parameters make it difficult to distinguish normal physiological changes from potential drug‐related toxicity.

Though some studies have shown a decrease in faecal calprotectin during pregnancy,^16^ FC levels consistently differentiate active from inactive disease, even during pregnancy. The current ECCO guideline states that faecal calprotectin can reliably monitor disease activity during pregnancy.^9^ Therefore, a faecal calprotectin concentration of >200 μg/g was considered a biochemical indicator of disease activity. Clinical disease activity was noted if the treating physician prescribed remission‐inducing medication (corticosteroids, budesonide, anti‐TNF agents) due to IBD‐related complaints.

For AZA/MP users, 6‐TGN concentrations within the range of 235–500 pmol/8 × 10^8^ RBC were considered therapeutic.^17^ For TG users, an upper therapeutic limit of 1000 pmol/8 × 10^8^ RBC was applied.^11^, ^18^, ^19^ 6‐MMPR levels over 5700 pmol/8 × 10^8^ RBC have been linked to hepatotoxicity,^20^ and were consequently noted as supratherapeutic levels. Currently, there are no reliable cut‐off values for 6‐TGN if the thiopurine is used as part of combination therapy.^21^ Lower targets have been suggested, as the thiopurine is then used to reduce the risk of immunogenicity rather than targeting remission, though no definitive evidence has been produced. All analyses will be adjusted for use as part of combination therapy.

Adverse pregnancy outcomes included low birthweight (LBW), defined as a birthweight of less than 2500 g; preterm birth, occurring when a pregnancy lasted less than 37 weeks; and dysmaturity (small for gestational age, SGA), indicated by a birthweight below the tenth percentile of expected weight with regard to the duration of pregnancy. All adverse outcomes were assessed separately.

### 2.4 Statistical analysis

#### 2.4.1 Descriptives

Baseline clinical characteristics were presented as numbers with percentages for categorical variables, means with standard deviations (SD) for normally distributed continuous parameters and medians with interquartile ranges (IQR) for skewed continuous parameters.

#### 2.4.2 Course of 6‐TGN and 6‐MMPR during pregnancy

First, linear mixed‐effects models (LMMs) were used to assess the course of 6‐TGN and 6‐MMPR measurements over time during pregnancy. In these models, time was categorized into intervals corresponding to the pre‐conceptional period (baseline), the first and second halves of each trimester (1–3) and the postpartum period, and was treated as a fixed effect. All measurements during pregnancy and the post‐partum period were compared to the baseline measurements. To account for repeated measurements, the subject ID was treated as a random effect. Further, the models were adjusted for dosage and the use of thiopurines as part of anti‐TNF combination therapy, by including both variables as fixed effects. As mesalamine has been suggested to increase 6‐TGN concentrations without affecting 6‐MMPR levels in some studies,^22^ we performed a sensitivity analysis excluding women who used concomitant mesalamine to assess whether this could have influenced our findings. Results were expressed as Estimated Marginal Means (EMM), accompanied by 95% confidence intervals and p‐values. The EMMs represent the estimated means of 6‐TGN and 6‐MMPR per time interval, adjusted for dosage, combination therapy and repeated measurements. The difference of EMMs between the timepoints and baseline is calculated and reported. Bonferroni adjustments were applied to adjust for multiple testing.

#### 2.4.3 Association between changes in metabolites and changes in disease activity and toxicity markers

Next, the absolute changes between baseline pre‐conceptional levels and samples taken during pregnancy were calculated for 6‐TGN and 6‐MMPR, and markers of disease activity (faecal calprotectin), hepatotoxicity (ALT) and myelotoxicity (leukocytes and platelets). Using the change of 6‐TGN and 6‐MMPR as fixed effects, the association between these values and the changes in calprotectin, ALT, leukocytes and platelets was examined using univariate LMMs correcting for repeated measurements. It is expected that the predictors (6‐TGN and 6‐MMPR) and outcomes (ALT, leukocytes and platelets) of this analysis will change considerably over the course of the pregnancy and following potential dosage alterations during pregnancy. Therefore, multivariate LMMs adjusting for time, dosage and anti‐TNF combination therapy as fixed effects were performed subsequently. It could be argued that modest changes in thiopurine metabolism may have less impact in women receiving combination therapy. For this reason, a sensitivity analysis restricted to women receiving thiopurine monotherapy was performed. The change in EMMs of the markers per unit increase in 6‐TGN and 6‐MMPR is reported, along with 95% confidence intervals and p‐values.

#### 2.4.4 Handling of missing data

All outcome markers were collected only if measured within 2 weeks before or after 6‐TGN and 6‐MMPR measurements. This approach ensured that no predictor values were missing, but may introduce bias, as women without 6‐TGN or 6‐MMPR measurements during these timepoints were excluded through list‐wise deletion. Baseline differences were therefore assessed between individuals with and without measurements at each timepoint.

Further, the absence of markers in women with 6‐TGN and 6‐MMPR measurements may have been influenced by clinical decision‐making. For example, liver values could be assessed more stringently in those with supratherapeutic drug levels, while calprotectin could be measured more often in those with subtherapeutic levels. We therefore examined patterns of absence for all markers using visual inspection (scatterplots and boxplots) and statistical analyses (t‐tests and logistic regression including timepoints and dosing as covariates). Furthermore, a sensitivity analysis was performed using multiple imputation. Missing values of markers were imputed five times, whereafter the changes from baseline levels to levels during pregnancy were calculated. The LMMs were then repeated using both the original data and the imputed datasets, and the pooled results were compared.

#### 2.4.5 Model specifications

In all LMMs, the maximum likelihood estimation method was used with 150 maximum iterations. In order to choose the best‐fitting covariance structure, the Akaike information criterion (AIC) was used. The normality assessment of the residuals was conducted visually using normal probability (Q‐Q) plots, and statistically using Shapiro–Wilk tests. Homoscedasticity was visually confirmed using scatterplots. If needed, the variables in the models were log‐transformed to achieve normality and/or homoscedasticity. To improve interpretability, outcomes after analyses using log‐transformed values were back‐transformed for visualization purposes. All analyses were performed using IBM SPSS statistics (V.28.0), and all figures were made using RStudio (2024.09.0 Build 375). *P*‐values of ≤0.05 were considered statistically significant.

### 2.5 Nomenclature of targets and ligands

Key protein targets and ligands in this article are hyperlinked to corresponding entries in http://www.guidetopharmacology.org, and are permanently archived in the Concise Guide to PHARMACOLOGY 2021/22.^23^

## 3 RESULTS

### 3.1 Population

A total of 87 women using thiopurines during pregnancy were included for analysis, comprising 100 pregnancies. Approximately two‐thirds of included women had CD (64.4%), while one third had UC (32.2%) and three women had IBD‐unclassified (IBD‐U) (3.4%). Either AZA or MP was used in 76 pregnancies (76.0%), while TG was used in 24 pregnancies (24.0%). In 26 pregnancies (26.0%), the thiopurine was used as part of combination therapy with anti‐TNF. All baseline characteristics can be found in Table 1.

**TABLE 1:** Patient characteristics at conception.

Maternal characteristics	*N* = 87
Inflammatory bowel disease subtype, *n* (% of cases):	
‐ Ulcerative colitis	28 (32.2)
‐ Crohn's disease	56 (64.4)
‐ IBD undetermined	3 (3.4)
Montreal classification UC, *n* (% of cases):	
E: extension of colitis:	
‐ E2: left‐sided colitis	6 (21.4)
‐ E3: extensive colitis	15 (53.6)
‐ Unknown	7 (25.0)
S: severity of colitis:	
‐ S1: Mild UC	4 (14.3)
‐ S2: Moderate UC	5 (17.9)
‐ S3: Severe UC	3 (10.7)
‐ Unknown	16 (57.1)
Montreal classification CD, *n* (% of cases):	
A: age at diagnosis	
‐ A1: (≤16 years)	8 (14.3)
‐ A2: (17–40 years)	45 (80.4)
‐ Unknown	3 (5.4)
L: disease location	
‐ L1: terminal ileum only	10 (17.9)
‐ L2: colon only	9 (16.1)
‐ L3: ileum and colon	33 (58.9)
‐ + L4: locations proximal to ileum	4 (7.1)
‐ + P: perianal disease	11 (19.6)
‐ Unknown	3 (5.4)
B: behaviour	
‐ B1: non‐stricturing, non‐penetrating	42 (75.0)
‐ B2: stricturing	7 (12.5)
‐ B3: penetrating	4 (7.1)
‐ Unknown	3 (5.4)

Pregnancy level characteristics	*N* = 100
Type of thiopurine *n* (% of cases)	
‐ Azathioprine	60 (60.0)
‐ Mercaptopurine	16 (16.0)
‐ Thioguanine	24 (24.0)
Dose (mg/kg), median (IQR)	
‐ Azathioprine	1.5 (0.9–2.2)
‐ Mercaptopurine	0.8 (0.5–1.0)
‐ Thioguanine	0.2 (0.1–0.3)
Used allopurinol prior to pregnancy, *n* (%)	7 (7.0)
Duration of thiopurine‐use prior to pregnancy (years), median (IQR)	3.7 (1.9–6.8)
Smoking status, *n* (% of cases)	
‐ Current	5 (5.0)
‐ Former (before pregnancy)	20 (20.0)
‐ Never	67 (67.0)
‐ Unknown	8 (8.0)
Body mass index prior to pregnancy (kg/m^2^), median (IQR)	23.7 (20.8–26.6)
Maternal age at conception (years), median (IQR)	31.3 (28.1–34.2)
Disease duration (years), median (IQR)	8.1 (5.0–11.5)
Gravidity (>1), *n* (% of cases)	42 (42.0)
IBD co‐medication at conception, *n* (% of cases)	40 (40.0)
‐ Aminosalicylates	14 (14.0)
‐ Corticosteroids, non‐systemic	1 (1.0)
‐ Corticosteroids, systemic	3 (3.0)
‐ TNF alpha inhibitors^a^	26 (26.0)
‐ Ustekinumab	2 (2.0)
‐ Vedolizumab	1 (1.0)

Abbreviations: IBD, Inflammatory bowel disease; UC, ulcerative colitis; CD, Crohn's disease; mg, milligram; kg, kilogram; IQR, interquartile range.

^a^ TNF alpha inhibitors include infliximab, adalimumab, golimumab.

In those using AZA or MP, 214 6‐TGN measurements and 214 6‐MMPR measurements were recorded. In those using TG, 69 6‐TGN measurements were logged. The number of observations per time interval can be found in Table S1.

### 3.2 Course of 6‐TGN and 6‐MMPR during pregnancy

At baseline, the back‐transformed EMM of 6‐TGN, corrected for dosage and repeated measurements, was 602.5 pmol/8 × 10^8^ RBC (95% CI: 468.7 to 773.6) based on 12 measurements in women using TG, and 261.7 pmol/8 × 10^8^ RBC (95% CI: 221.6 to 308.6) based on 31 measurements in those using AZA or MP. For 6‐MMPR, the EMM prior to pregnancy in women using AZA or MP (based on 31 measurements) was 1370.6 pmol/8 × 10^8^ RBC (95% CI: 1006.3 to 1865.0).

A statistically significant decrease in 6‐TGN was observed from the first half of the second trimester (EMM difference −0.357, 95% CI ‐0.6 to −0.1, p = 0.001) up until the first half of the third trimester (EMM difference −0.335, 95% CI ‐0.6 to −0.1, p = 0.007) in women using AZA or MP, based on log‐transformed values. 6‐TGN did not fully return to baseline levels in the postpartum period, as defined in this study (six months after birth). In women using TG, a significant decrease in 6‐TGN compared to pre‐pregnancy values was observed in the second half of the third trimester (EMM difference −0.396, 95% CI ‐0.8 to 0.0, p = 0.027). These findings reflect a statistically significant decrease in group‐level estimated means and do not indicate a uniform decline across all individuals.

**FIGURE 1:**
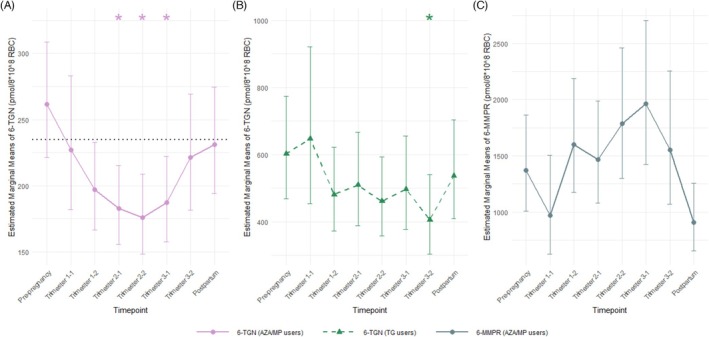
Estimated marginal means of metabolite concentrations per timepoint. Estimated marginal means (EMM) per timepoint. The error bars represent the 95% confidence interval. Values that differ significantly from the baseline pre‐pregnancy measurements are marked with an asterisk. These values were back‐transformed from a log‐linear model to improve interpretability. 6‐TGN levels were obtained from women treated with either azathioprine, mercaptopurine or thioguanine. The analysis on 6‐MMPR was performed in women using either azathioprine or mercaptopurine only. The dotted line indicates the therapeutic threshold. (A) 6‐TGN in women using azathioprine or mercaptopurine. (B) 6‐TGN in women using thioguanine. (C) 6‐MMPR in women using azathioprine or mercaptopurine.

In contrast, 6‐MMPR numerically increased during pregnancy, particularly in the second half of the second trimester (EMM difference 0.265, 95% CI ‐0.3 to 0.8, p = 1.000) and the first half of the third trimester (EMM difference 0.359, 95% CI ‐0.2 to 0.9, p = 1.000). However, after Bonferroni correction for multiple comparisons, none of these differences were statistically significant (adjusted p‐values = 1.000). A sensitivity analysis excluding women on concomitant amino salicylates (14%) showed similar trajectories for both 6‐TGN and 6‐MMPR levels across pregnancy, suggesting that amino salicylates did not materially influence the observed metabolite changes. The outcomes of the main analyses are depicted in Figure 1. The complete outcome for the entire cohort and for the analysis excluding women using amino salicylates can be found in Tables S1 and S2.

### 3.3 Association between 6‐TGN/6‐MMPR and markers of efficacy and safety

In the 56 pregnancies of 47 women using AZA or MP as monotherapy, 37 pregnancies (78.7%) had at least one subtherapeutic 6‐TGN measurement. Across the entire cohort, biochemical evidence of active disease was present at the time of 43 metabolite measurements across 33 pregnancies (33.0%) in 32 women. Clinical disease activity was noted in 13 pregnancies (13.0%) and coincided with nine metabolite measurements. In the linear mixed models (LMMs), 6‐TGN showed a negative, though statistically non‐significant, association with faecal calprotectin for both AZA/MP users (EMM difference −1.311, 95% CI ‐3.8 to 1.2, p = 0.287) and TG‐users (EMM difference −0.829, 95% CI ‐2.2 to 0.5, p = 0.214). The sensitivity analysis restricted to women receiving thiopurine monotherapy was performed, showing similar non‐significant associations between changes in 6‐TGN and calprotectin. In women on TG monotherapy, the multivariate analysis yielded an EMM difference of −2.046 (95% CI ‐4.6 to 0.5, p = 0.100), and in women on AZA/MP monotherapy, the EMM difference was −0.589 (95% CI ‐2.1 to 0.9, p = 0.420).

Supratherapeutic levels of 6‐TGN were recorded five times (5.0% of pregnancies), while supratherapeutic 6‐MMPR levels were observed 28 times (21.0% of pregnancies) during pregnancy. Co‐medication was not adjusted following supratherapeutic measurements, though thiopurine dose modifications were frequently made.

Supratherapeutic 6‐TGN levels were relatively rare and mostly occurred in isolation. These measurements led to a dose reduction in one case. In the other cases (n = 4, 4.0%), the levels decreased spontaneously or were not measured again.

Elevated 6‐MMPR levels were frequently observed during pregnancy, often exceeding 8000 pmol/8 × 10^8^ RBC (n = 14, 50.0% of all supratherapeutic measurements). Dose adjustments were not uniformly applied. In many instances, supratherapeutic 6‐MMPR values did not lead to intervention (n = 19, 67.9%), particularly when corresponding 6‐TGN levels were low (n = 5, 17.9%), or when spontaneous decreases were noted in subsequent measurements (n = 14, 50.0%). Dose reductions, when undertaken, were usually prompted by persistently high or rising 6‐MMPR concentrations or by changes in TGN levels rather than a strict numerical threshold alone. A marked postpartum decline in 6‐MMPR was a consistent pattern, often following dose reductions initiated late in pregnancy.

There were two instances of transient leukopenia during pregnancy, in which leukocyte counts normalized without any dosage adaptations. In another case, a leukopenia of 2.4*10^9^ L^−1^ was found in the first trimester. Azathioprine was discontinued, leading to normalization of the leukocyte count. One pregnancy was complicated by transient thrombocytopenia, and in two others, platelet counts of 106 × 10^9^ L^−1^ and 100 × 10^9^ L^−1^ were recorded, though no further measurements were taken.

Liver enzyme elevations were observed in nine pregnancies, with three cases exceeding twice the upper limit of normal for ALT or AST. Most elevations were modest and either resolved during pregnancy (n = 3) or declined postpartum (n = 3) without any dose adaptation. Some cases lacked follow‐up despite elevated values. None of the liver enzyme elevations led to clinical intervention. Dose adjustments were occasionally made based on metabolite levels rather than liver enzymes, and one patient was switched from MP to TG due to high 6‐MMPR concentrations.

In the LMMs among women using TG, increases in 6‐TGN were correlated with a modest increase in leukocyte counts (EMM difference 0.004, 95% CI: −0.1 to 0.1, p = 0.015). Further, increases in 6‐MMPR were significantly associated with increases in ALT in women using AZA or MP, although the effect size was small (EMM difference 0.002, 95% CI: 0.0 to 0.0, p < 0.001).

No further significant associations were identified between changes in 6‐TGN and 6‐MMPR from preconception to pregnancy and changes in any of the efficacy or safety markers. The medians of the investigated markers and metabolites are depicted in Figures 2A and 2B. The full outcome of the LMMs can be found in Table S3.

**FIGURE 2a:**
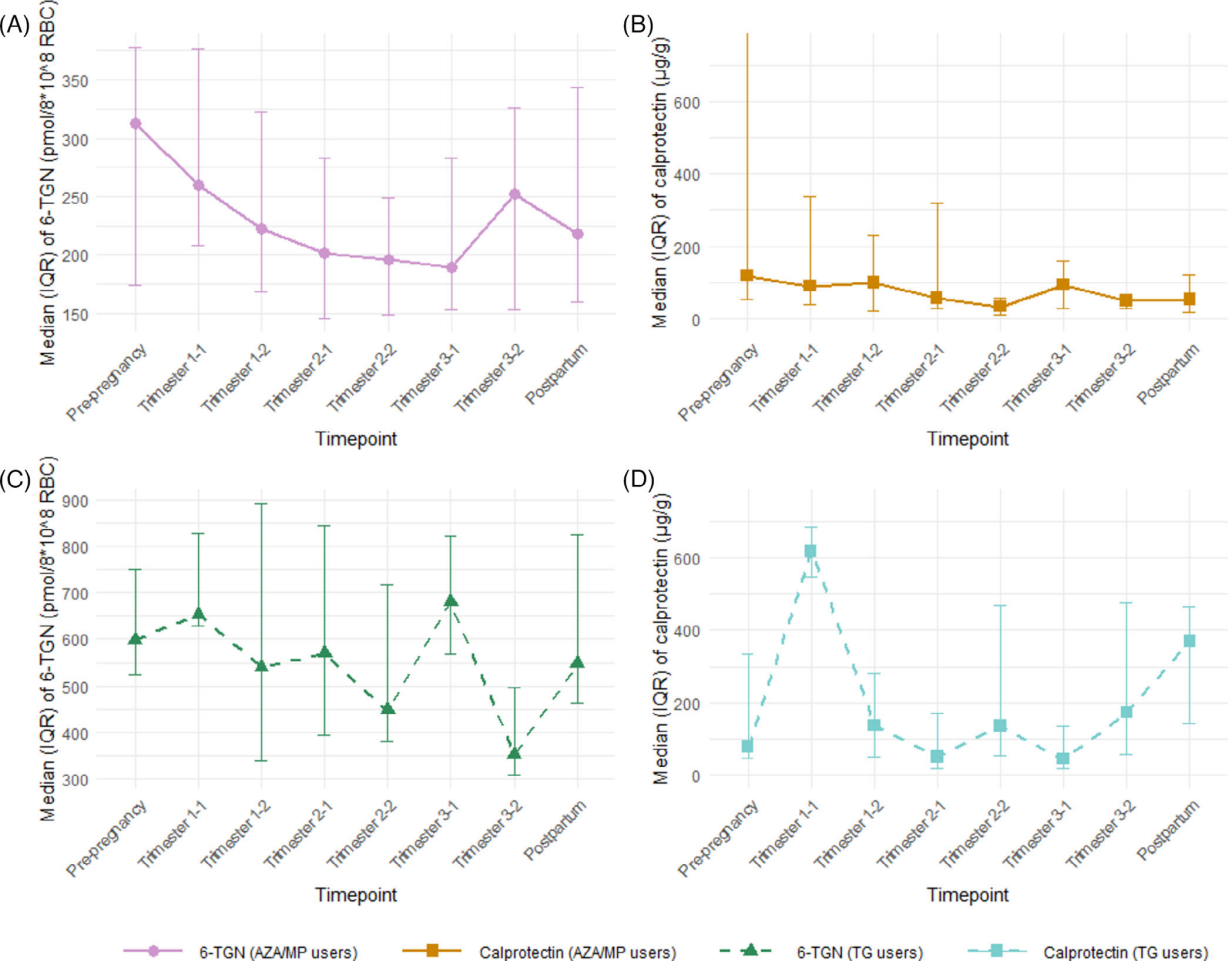
Median 6‐TGN and faecal calprotectin concentrations per timepoint. Median of all measured values per timepoint. The error bars represent the IQR. (A) 6‐TGN in women using azathioprine or mercaptopurine. (B) Faecal calprotectin in women using azathioprine or mercaptopurine. (C) 6‐TGN in women using thioguanine. (D) Faecal calprotectin in women using thioguanine.

**FIGURE 2b:**
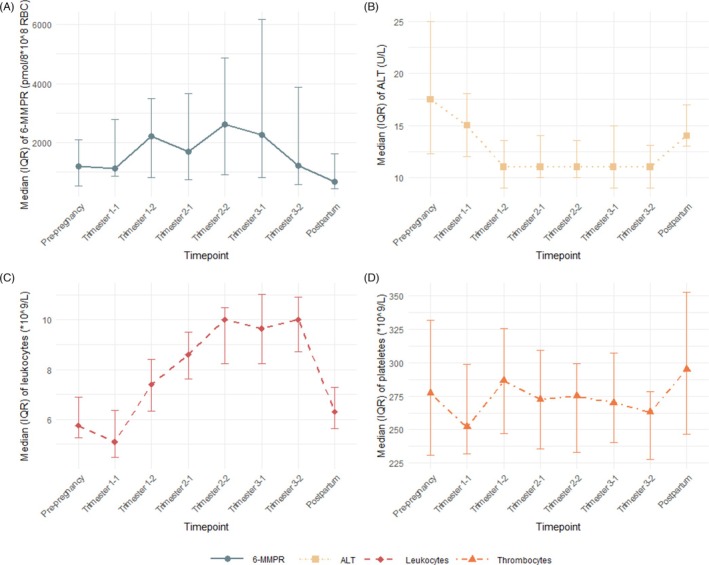
Median 6‐MMPR concentrations, ALT, leukocyte and platelets per timepoint in women using azathioprine or mercaptopurine. Median of all measured values per timepoint. The error bars represent the IQR. Figure 2 b includes outcomes of women using either azathioprine or mercaptopurine, not thioguanine. (A) 6‐MMPR. (B) ALT. (C) Leukocytes. (D) Platelets.

### 3.4 Missing data

With the exception of a higher proportion of women with IBD‐U among those with metabolite measurements in the first half of the third trimester compared to those without (7.5% *vs*. 0.0%, p = 0.031), there were no significant baseline differences in age, smoking status, phenotype, disease duration, or use of combination therapy at any timepoint. Full results for these comparisons are available in Table S4. Absence of calprotectin, ALT, leukocytes and platelets was not significantly associated with 6‐TGN levels in any tests. There was a significant difference in 6‐MMPR between those in whom calprotectin was missing *vs*. those in whom it was present, according to the t‐test (p = 0.038). However, in the logistic regression analysis, the trimester variable was significantly associated with absence of calprotectin (p = 0.042), and not 6‐MMPR (p = 0.108), suggesting that the likelihood of missing calprotectin data may vary depending on the timepoint in the pregnancy, rather than being directly influenced by the levels of 6‐MMPR. Further, in the logistic regression analysis for 6‐MMPR, dosing was found to be significantly associated with the absence of thrombocyte data.

Visual inspection of scatterplots and boxplots revealed no discernible patterns between marker absence and 6‐TGN or 6‐MMPR levels, suggesting that the probability of missing data was not systematically related to drug levels. In the sensitivity analyses using imputed datasets, the pooled results were consistent with the findings of the primary analysis. No substantial differences in the direction or magnitude of effect estimates were noted. In summary, missing data appeared largely unrelated to clinical characteristics or metabolite levels, and sensitivity analyses confirmed the robustness of the primary findings, supporting the assumption that the observed absence of markers did not substantially bias the results.

### 3.5 Pregnancy‐outcomes

None of the pregnancies ended in pregnancy loss before the sixteenth week. An adverse pregnancy outcome was noted in 13 pregnancies (13.0%). These adverse outcomes consisted of prematurity in seven cases (7.0%), low birth weight in four cases (4.0%) and dysmaturity in three cases (3.0%). Two pregnancies (2.0%) were complicated by intrahepatic cholestasis of pregnancy (ICP).

## 4 DISCUSSION

In this retrospective multicentre cohort study of pregnant women with IBD, we found no evidence that changes in thiopurine metabolite levels during pregnancy were associated with clinically meaningful changes in markers of efficacy or safety. Faecal calprotectin levels remained largely stable throughout pregnancy, and hepatotoxicity and myelosuppression were uncommon. While an inverse relationship between changes in 6‐TGN and faecal calprotectin was observed, this association was not statistically significant. The fluctuations in 6‐TGN and 6‐MMPR levels previously described in literature were largely reflected in our cohort.^6^, ^7^

A statistically significant decrease of the active 6‐TGN metabolites was observed in the second and third trimester in AZA/MP‐users, and in the third trimester in TG‐users. There was no significant difference between pre‐conceptional 6‐MMPR levels and 6‐MMPR measurements during pregnancy and postpartum. In previous studies, more notable changes in 6‐MMPR/6‐TGN ratios and 6‐MMPR have been reported.^6^, ^7^, ^8^ In the present study, a different statistical method has been employed to analyse the measurements over time. Rather than comparing median values, LMMs were used to calculate the EMM. This approach allowed for adjustment for dosage, repeated measurements and inter‐individual variability, meaning the outcomes may reflect the true variation of both 6‐TGN and 6‐MMPR during pregnancy more accurately. According to the current knowledge, we cannot explain the mechanism of our observation. It is likely multifactorial and may involve pregnancy‐related changes in thiopurine‐metabolizing enzymes, intracellular transport and/or erythrocyte turnover. Given the limited and often tissue‐specific data available, further mechanistic studies are needed.

When focusing on efficacy markers in relation to the established decrease of 6‐TGN during pregnancy, an inverse, statistically non‐significant relation with changes in faecal calprotectin was found for both AZA/MP and TG‐users. In accordance with a recent study,^8^ our results seem to indicate that the decrease of 6‐TGN is only modestly associated with faecal calprotectin. Although clinical disease activity markers (e.g. physician global assessment) were not available, the known correlation between calprotectin and clinical disease activity during pregnancy^24^, ^25^ suggests that the small effect of 6‐TGN on calprotectin likely reflects a limited clinical impact.

When focusing on safety markers, a statistically significant, but probably clinically irrelevant, positive correlation was observed between changes in 6‐MMPR and changes in ALT. It is known that ALT decreases slightly during pregnancy,^14^ which could mask some of the potential positive association between 6‐MMPR and ALT. Similar to pregnancies in healthy women and women with IBD not using a thiopurine, an increase of ALT during pregnancy in this population should always be considered pathological and should prompt further investigation. This is especially important considering recent findings showing women using thiopurine during pregnancy have a higher risk of developing ICP.^8^, ^26^, ^27^, ^28^, ^29^

Pregnancy outcomes in this cohort, as well as in previous studies on thiopurine^6^, ^7^, ^8^ and thioguanine^30^ use, were comparable to outcomes in the general IBD population^31^, ^32^ and the healthy Dutch population.^33^ 6‐TGN passively crosses the placenta,^34^ with infant levels at birth strongly correlated with, but lower than, maternal levels.^6^, ^7^ Low levels of 6‐MMPR have also been detected in infants, though much lower than maternal levels.^6^, ^7^, ^34^ Intrauterine exposure can occasionally cause adverse effects such as anaemia or severe neutropenia, often linked to maternal supratherapeutic 6‐TGN levels, though not exclusively. Thrombocytosis and abnormal liver tests have also been reported without detectable infant metabolite levels.^7^ Exposed infants clear these metabolites by six weeks.^7^ In our cohort, no such adverse effects were reported to the treating physicians; however, infant outcomes such as haematological or hepatic abnormalities were not systematically evaluated, and umbilical cord blood samples were not collected. Therefore, no firm conclusions can be drawn regarding neonatal safety in this study.

It has been shown that disease activity and toxicity in the non‐pregnant population may relate to decreases in 6‐TGN and increases in 6‐MMPR concentrations, respectively.^35^, ^36^ These findings have led to the discourse focusing on the usefulness of therapeutic drug monitoring of thiopurines in all patients with IBD.^35^, ^36^, ^37^, ^38^, ^39^ Consequently, brought about by the finding of fluctuating 6‐TGN and 6‐MMPR levels during pregnancy,^6^, ^7^ this led to the advice to monitor drug levels and alter the dosage if levels change during pregnancy.^9^ However, it is possible that sustained altered drug levels must exist for a prolonged period of time to effectively influence disease activity and toxicity. This would reflect clinical practice, where a slow onset of action is seen, and therapy withdrawal is linked to relapse, although not immediately.^40^, ^41^ Based on our results, the correlation between metabolite concentrations and clinical outcomes may not be as evident during the relatively short duration of pregnancy.

If the alterations of 6‐TGN and 6‐MMPR are only associated with limited, mostly non‐significant effects on markers of efficacy and safety, the benefit of dose adjustments following the measurement of altered drug levels becomes uncertain. Furthermore, the impact of dose adjustments based on metabolite levels remains unpredictable due to high intra‐ and inter‐individual variability,^38^, ^39^ and dose increases have previously been associated with elevated levels of 6‐MMPR and risk of ICP in late pregnancy.^8^

Hence, it could be argued that the proposed monitoring of metabolites during pregnancy is not warranted in all patients in order to achieve better clinical outcomes in terms of disease activity, toxicity and pregnancy. It may be considered to measure metabolites only in women who exhibit signs of disease activity or toxicity based on routine IBD symptom monitoring and laboratory tests, including complete blood count and liver enzymes, during pregnancy.

This study has several notable strengths. First, to our knowledge, this study included the largest sample size and number of measurements to date in research on thiopurine metabolite levels during pregnancy in IBD patients, enhancing the reliability and generalizability of the findings. Additionally, the use of LMMs allowed for adjustment for repeated measurements and dosage, providing a more accurate assessment of the relationships between variables over time.

There are a few important limitations to consider. First, certain confounders—particularly phenotype, periconceptional flares and biologic use—were not included in the linear mixed models to avoid overfitting, although they may influence disease activity during pregnancy^42^, ^43^ Pharmacogenetic testing was not routinely performed, so undiagnosed TPMT or NUDT15 variants cannot be fully excluded, though all patients were on stable thiopurine therapy prior to pregnancy. Direct measures of adherence were unavailable, and non‐adherence during pregnancy cannot be fully excluded. Thiopurine metabolites were measured at the discretion of the treating physician, introducing potential selection bias, although no major baseline differences were observed between patients with and without measurements. Further, apart from allopurinol, other potential interacting drugs, such as ribavirin or vitamin K antagonists, were not recorded. Lastly, as mentioned previously, disease activity indices were unfortunately not available at baseline or during pregnancy, as they were not routinely documented.

In conclusion, while 6‐TGN metabolite concentrations decreased during pregnancy, these fluctuations were not associated with meaningful alterations in disease activity or toxicity markers. Our findings suggest that routine proactive monitoring of thiopurine metabolites during pregnancy offers limited clinical utility and is unnecessary in most patients. Instead, a more pragmatic and targeted strategy—emphasizing clinical evaluation and standard laboratory monitoring of disease activity and toxicity—appears sufficient for guiding treatment during pregnancy. Future larger, prospective studies are warranted to validate these findings and to further optimize thiopurine management in this population.

## AUTHOR'S CONTRIBUTIONS

D.G.B. Planning; conducting the study; collecting and interpreting data; statistical analysis; drafting the manuscript; approval of the final manuscript.

P.M. Study concept and design; study supervision; critical revision; approval of the final manuscript.

F.G. Collecting and interpreting data; critical revision; approval of the final manuscript.

D.v.d.B.‐Z. Collecting and interpreting data; critical revision; approval of the final manuscript.

A.E.v.d.M. Collecting and interpreting data; critical revision; approval of the final manuscript.

F.C. Collecting and interpreting data; critical revision; approval of the final manuscript.

K.H.N.d.B. Collecting and interpreting data; critical revision; approval of the final manuscript.

D.S.D. Collecting and interpreting data; critical revision; approval of the final manuscript.

D.R.W. Collecting and interpreting data; critical revision; approval of the final manuscript.

L.J.J.D. Collecting and interpreting data; critical revision; approval of the final manuscript.

G.D. Study supervision; critical revision; approval of the final manuscript.

A.R.B. Statistical analysis; critical revision; approval of the final manuscript.

C.J.v.d.W. Study supervision; critical revision; approval of the final manuscript.

M.C.V. Study concept and design; study supervision; critical revision; approval of the final manuscript.

## CONFLICT OF INTEREST STATEMENT

D.G.B. received speaker fees from Takeda and Galapagos.

P.M. none to declare.

F.G. none to declare.

D.v.d.B‐Z none to declare.

A.E.v.d.M. received research grants from Galapagos, Abbvie, Ferring, ZonMW.

K.H.N.d.B. has served as a speaker for AbbVie and MSD and has served as a consultant and principal investigator for TEVA Pharma BV and Takeda. He has received a research grant (unrestricted) from Dr. Falk, TEVA Pharma BV, Dutch Digestive Foundation (MLDS) and Takeda. All outside the submitted work.

D.S.D. none to declare.

D.R.W. none to declare.

L.J.J.D has served as a speaker for AbbVie, Celltrion, Janssen‐Cilag and Takeda and has developed continuing education materials for Ferring, all outside the submitted work.

G.D. received research grants from Royal DSM and Janssen Pharmaceuticals, advisory board fee from Pharmacosmos and ASTRA‐ZENECA, and speakers fee from Abbvie.

A.R.B. received a research grant from Janssen Pharmaceuticals and received speaker's fees from AbbVie and Ferring, outside the submitted work.

C.J.v.d.W. received grants from ZonMW, Falk and Pfizer, has received consulting fees from Janssen, Galapagos, and Pfizer, has received payment or honoraria for lectures, presentations, speakers bureaus, manuscript writing or educational events from Ferring and AbbVie, and had leadership roles in the European Crohn's & Colitis organization, United European Gastroenterology council and the Dutch Association for Gastroenterology (NVGE).

M.C.V. received speaker fees from Janssen‐Cilag, Galapagos, and Ferring B.V.

## Supporting information


**Table S1.** Estimated marginal mean differences in log‐transformed values compared to pre‐pregnancy levels^†^

**Table S2.** Estimated marginal mean differences in log‐transformed values compared to pre‐pregnancy levels in women not using amino salicylates^†^

**Table S3.** Associations between changes in metabolite concentrations and markers for disease‐activity, hepatotoxicity and myelotoxicity, adjusted for repeated measurements.

**Table S4.** Baseline differences between patients with metabolite measurements *vs.* patients without metabolite measurements at each timepoint.

## Data Availability

The data underlying this article cannot be shared publicly in order to protect the privacy of individuals that participated in the study. The data will be shared on reasonable request to the corresponding author.
