# Supplementary material for: The decline of 6‐thioguanine nucleotides is not linked to impaired efficacy or safety of thiopurines in pregnant women with inflammatory bowel disease
Source: Br J Clin Pharmacol. 2026 Mar 18;92(7):2364–76. doi: 10.1002/bcp.70520 (PMC13304283; doi:10.1002/bcp.70520)
Supplement: Supplementary file 1 — Table S1. Estimated marginal mean differences in log‐transformed values compared to pre‐pregnancy levels† [file BCP-92-2364-s002.docx]

**Supplements**

**Supplementary table 1.** Estimated marginal mean differences in log-transformed values compared to pre-pregnancy levels^†^

| Timepoint^‡^ | Observations | EMM difference | 95% CI Lower | 95% CI Higher | p-value |
| --- | --- | --- | --- | --- | --- |
| Course of 6-TGN during pregnancy in women using thioguanine | | | | | |
| Trimester 1 -1 | 3 | 0.072 | -0.456 | 0.599 | 1.000 |
| Trimester 1 -2 | 10 | -0.225 | -0.544 | 0.094 | 0.647 |
| Trimester 2 -1 | 8 | -0.169 | -0.532 | 0.193 | 1.000 |
| Trimester 2 -2 | 11 | -0.268 | -0.593 | 0.057 | 0.243 |
| Trimester 3 -1 | 9 | -0.192 | -0.597 | 0.213 | 1.000 |
| Trimester 3 -2 | 8 | -0.396 | -0.769 | -0.024 | **0.027** |
| Postpartum | 8 | -0.116 | -0.476 | 0.245 | 1.000 |
| Course of 6-TGN during pregnancy in women using azathioprine or mercaptopurine | | | | | |
| Trimester 1 -1 | 13 | -0.140 | -0.507 | 0.228 | 1.000 |
| Trimester 1 -2 | 31 | -0.283 | -0.567 | 0.001 | 0.052 |
| Trimester 2 -1 | 31 | -0.357 | -0.625 | -0.089 | **0.001** |
| Trimester 2 -2 | 27 | -0.396 | -0.675 | -0.117 | **<0.001** |
| Trimester 3 -1 | 30 | -0.335 | -0.619 | -0.051 | **0.007** |
| Trimester 3 -2 | 20 | -0.168 | -0.500 | 0.164 | 1.000 |
| Postpartum | 31 | -0.124 | -0.404 | 0.156 | 1.000 |
| Course of 6-MMPR during pregnancy in women using azathioprine or mercaptopurine | | | | | |
| Trimester 1 -1 | 12 | -0.344 | -1.098 | 0.409 | 1.000 |
| Trimester 1 -2 | 31 | 0.156 | -0.400 | 0.712 | 1.000 |
| Trimester 2 -1 | 31 | 0.068 | -0.468 | 0.604 | 1.000 |
| Trimester 2 -2 | 27 | 0.265 | -0.289 | 0.819 | 1.000 |
| Trimester 3 -1 | 30 | 0.359 | -0.204 | 0.923 | 1.000 |
| Trimester 3 -2 | 20 | 0.126 | -0.529 | 0.780 | 1.000 |
| Postpartum | 30 | -0.414 | -0.977 | 0.148 | 0.571 |

Abbreviations: EMM, estimated marginal mean; CI, confidence interval

† - Log-transformed values represent a multiplicative change in the outcome for each unit increase in the predictor. Here, each EMM difference corresponds to the factor by which the outcome is multiplied in each timepoint.

‡ - The timepoints were defined as follows: trimester 1-1 (up to day 45), trimester 1-2 (day 46 – day 91), trimester 2-1 (day 92 – day 141), trimester 2-2 (day 142 – day 189), trimester 3-1 (day 190 – day 235), trimester 3-2 (day 236 – birth), postpartum (up to six months after birth).
