# Supplementary material for: The decline of 6‐thioguanine nucleotides is not linked to impaired efficacy or safety of thiopurines in pregnant women with inflammatory bowel disease
Source: Br J Clin Pharmacol. 2026 Mar 18;92(7):2364–76. doi: 10.1002/bcp.70520 (PMC13304283; doi:10.1002/bcp.70520)
Supplement: Supplementary file 2 — Table S2. Estimated marginal mean differences in log‐transformed values compared to pre‐pregnancy levels in women not using amino salicylates† [file BCP-92-2364-s004.docx]

**Supplements**

**Supplementary table 2.** Estimated marginal mean differences in log-transformed values compared to pre-pregnancy levels in women not using amino salicylates^†^

| Timepoint^‡^ | Observations | EMM difference | 95% CI Lower | 95% CI Higher | p-value |
| --- | --- | --- | --- | --- | --- |
| Course of 6-TGN during pregnancy in women using thioguanine | | | | | |
| Trimester 1 -1 | 3 | 0.086 | -0.421 | 0.592 | 1.000 |
| Trimester 1 -2 | 9 | -0.203 | -0.529 | 0.124 | 1.000 |
| Trimester 2 -1 | 7 | -0.220 | -0.590 | 0.149 | 1.000 |
| Trimester 2 -2 | 9 | -0.330 | -0.670 | 0.010 | 0.066 |
| Trimester 3 -1 | 6 | -0.001 | -0.525 | 0.523 | 1.000 |
| Trimester 3 -2 | 7 | -0.417 | -0.797 | -0.037 | **0.020** |
| Postpartum | 8 | -0.105 | -0.461 | 0.251 | 1.000 |
| Course of 6-TGN during pregnancy in women using azathioprine or mercaptopurine | | | | | |
| Trimester 1 -1 | 10 | -0.138 | -0.569 | 0.293 | 1.000 |
| Trimester 1 -2 | 27 | -0.243 | -0.568 | 0.082 | 0.515 |
| Trimester 2 -1 | 26 | -0.298 | -0.605 | 0.009 | 0.067 |
| Trimester 2 -2 | 21 | -0.397 | -0.718 | -0.075 | **0.004** |
| Trimester 3 -1 | 24 | -0.335 | -0.664 | -0.007 | **0.041** |
| Trimester 3 -2 | 13 | -0.146 | -0.573 | 0.281 | 1.000 |
| Postpartum | 27 | -0.116 | -0.435 | 0.203 | 1.000 |
| Course of 6-MMPR during pregnancy in women using azathioprine or mercaptopurine | | | | | |
| Trimester 1 -1 | 10 | -0.259 | -1.088 | 0.571 | 1.000 |
| Trimester 1 -2 | 27 | 0.199 | -0.387 | 0.785 | 1.000 |
| Trimester 2 -1 | 26 | 0.137 | -0.433 | 0.708 | 1.000 |
| Trimester 2 -2 | 21 | 0.284 | -0.308 | 0.877 | 1.000 |
| Trimester 3 -1 | 24 | 0.379 | -0.225 | 0.983 | 1.000 |
| Trimester 3 -2 | 13 | 0.268 | -0.505 | 1.042 | 1.000 |
| Postpartum | 27 | -0.455 | -1.049 | 0.139 | 0.443 |

Abbreviations: EMM, estimated marginal mean; CI, confidence interval

† - Log-transformed values represent a multiplicative change in the outcome for each unit increase in the predictor. Here, each EMM difference corresponds to the factor by which the outcome is multiplied in each timepoint.

‡ - The timepoints were defined as follows: trimester 1-1 (up to day 45), trimester 1-2 (day 46 – day 91), trimester 2-1 (day 92 – day 141), trimester 2-2 (day 142 – day 189), trimester 3-1 (day 190 – day 235), trimester 3-2 (day 236 – birth), postpartum (up to six months after birth).
