# Supplementary material for: The decline of 6‐thioguanine nucleotides is not linked to impaired efficacy or safety of thiopurines in pregnant women with inflammatory bowel disease
Source: Br J Clin Pharmacol. 2026 Mar 18;92(7):2364–76. doi: 10.1002/bcp.70520 (PMC13304283; doi:10.1002/bcp.70520)
Supplement: Supplementary file 3 — Table S3. Associations between changes in metabolite concentrations and markers for disease‐activity, hepatotoxicity and myelotoxicity, adjusted for repeated measurements. [file BCP-92-2364-s003.docx]

**Supplements**

**Supplementary Table 3.** Associations between changes in metabolite concentrations and markers for disease-activity, hepatotoxicity, and myelotoxicity, adjusted for repeated measurements

|  |  | Univariate linear mixed model | | | Multivariate linear mixed model^†^ | | |
| --- | --- | --- | --- | --- | --- | --- | --- |
| Marker^‡^ | # | EMM difference^§^ | 95% CI | p-value | EMM difference^§^ | 95% CI | p-value |
| 6-TGN in women using thioguanine | | | | | | | |
| Calprotectin | 22 | -0.818 | -1.8 – 0.2 | 0.097 | -0.829 | -2.2 – 0.5 | 0.214 |
| ALT | 24 | 0.002 | -0.1 – 0.1 | 0.929 | 0.000 | -0.1 – 0.1 | 0.993 |
| Leukocytes | 24 | -0.001 | -0.0 – 0.0 | 0.755 | 0.004 | 0.0 – 0.0 | **0.015** |
| Platelets | 27 | 0.037 | -0.0 – 0.1 | 0.304 | 0.012 | -0.1 – 0.1 | 0.822 |
| 6-TGN in women using azathioprine or mercaptopurine | | | | | | | |
| Calprotectin | 38 | -0.819 | -3.1 – 1.5 | 0.470 | -1.311 | -3.8 – 1.2 | 0.287 |
| ALT | 57 | 0.004 | -0.1 – 0.1 | 0.889 | -0.006 | -0.1 – 0.1 | 0.835 |
| Leukocytes | 54 | -0.005 | -0.0 – 0.0 | 0.096 | -0.004 | -0.0 – 0.0 | 0.163 |
| Platelets | 51 | 0.052 | -0.1 – 0.2 | 0.466 | 0.078 | -0.1 – 0.2 | 0.255 |
| 6-MMPR in women using azathioprine or mercaptopurine | | | | | | | |
| Calprotectin | 55 | -0.016 | -0.1 – 0.0 | 0.520 | -0.016 | -0.1 – 0.0 | 0.578 |
| ALT | 65 | 0.002 | 0.0 – 0.0 | **0.003** | 0.002 | 0.0 – 0.0 | **<0.001** |
| Leukocytes | 63 | 7.515*10^-5^ | -8.9*10^-5^ – 0.0 | 0.361 | 5.675*10^-6^ | -0.0 – 0.0 | 0.952 |
| Platelets | 50 | -1.045*10^-5^ | -0.0 – 0.0 | 0.995 | 0.001 | -0.0 – 0.0 | 0.584 |

Abbreviations: #, number of observations; EMM, estimated marginal mean; CI, confidence interval; ALT, alanine transaminase

† - Model adjusted for dosage, anti-TNF co-medication and trimester.

‡ - The absolute change in the value of the markers between pre-conceptional baseline measurements and any timepoint during pregnancy.

§ - Represents the effect of a unit increase in the values of 6-TGN or 6-MMPR on the outcome variable.
