# Supplementary material for: The decline of 6‐thioguanine nucleotides is not linked to impaired efficacy or safety of thiopurines in pregnant women with inflammatory bowel disease
Source: Br J Clin Pharmacol. 2026 Mar 18;92(7):2364–76. doi: 10.1002/bcp.70520 (PMC13304283; doi:10.1002/bcp.70520)
Supplement: Supplementary file 4 — Table S4. Baseline differences between patients with metabolite measurements vs. patients without metabolite measurements at each timepoint. [file BCP-92-2364-s001.docx]

**Supplements**

**Supplementary Table 4.** Baseline differences between patients with metabolite measurements versus patients without metabolite measurements at each timepoint

| **Characteristic** | **Measurement present** | **No measurements** | **p-value** |
| --- | --- | --- | --- |
| Pre-pregnancy   - Age (y), median (IQR) - Active smoking, n (%) - IBD phenotype, n (%)   - CD - UC - IBD-U   - Disease duration (y), median (IQR) - Biologic therapy, n (%) | 31.3 (26.6 – 33.4)  3 (7.1)  27 (64.3)  13 (31.0)  2 (4.8)  7.6 (4.4 – 11.6)  14 (33.3) | 31.5 (28.9 – 34.7)  2 (3.5)  38 (65.5)  19 (32.8)  1 (1.7)  8.3 (4.9 – 11.7)  13 (22.4) | 0.261  0.403  0.899  0.848  0.379  0.839  0.225 |
| Trimester 1-1^†^   - Age (y), median (IQR) - Active smoking, n (%) - IBD phenotype, n (%)   - CD - UC - IBD-U   - Disease duration (y), median (IQR) - Biologic therapy, n (%) | 32.4 (31.2 – 34.5)  0 (0.0)  9 (56.3)  7 (43.8)  0 (0.0)  5.2 (2.7 – 10.6)  5 (31.3) | 31.1 (27.7 – 33.8)  5 (6.0)  56 (66.7)  25 (29.8)  3 (3.6)  8.4 (5.2 – 12.4)  22 (26.2) | 0.100  0.317  0.423  0.272  0.443  0.062  0.676 |
| Trimester 1-2^†^   - Age (y), median (IQR) - Active smoking, n (%) - IBD phenotype, n (%)   - CD - UC - IBD-U   - Disease duration (y), median (IQR) - Biologic therapy, n (%) | 31.7 (28.0 – 34.8)  3 (7.5)  24 (60.0)  15 (37.5)  1 (2.5)  8.5 (5.4 – 10.8)  9 (22.5) | 31.3 (28.5 – 33.4)  2 (3.3)  41 (68.3)  17 (28.3)  2 (3.3)  7.2 (3.9 – 12.6)  18 (30.0) | 0.368  0.349  0.392  0.336  0.811  0.627  0.408 |
| Trimester 2-1^†^   - Age (y), median (IQR) - Active smoking, n (%) - IBD phenotype, n (%)   - CD - UC - IBD-U   - Disease duration (y), median (IQR) - Biologic therapy, n (%) | 31.8 (28.5 – 34.9)  1 (2.7)  24 (64.9)  13 (35.1)  0 (0.0)  7.3 (4.5 – 11.8)  11 (29.7) | 31.2 (27.9 – 33.7)  4 (6.4)  41 (65.1)  19 (30.2)  3 (4.8)  8.2 (4.9 – 11.5)  16 (25.4) | 0.289  0.419  0.983  0.607  0.178  0.972  0.637 |
| Trimester 2-2^†^   - Age (y), median (IQR) - Active smoking, n (%) - IBD phenotype, n (%)   - CD - UC - IBD-U   - Disease duration (y), median (IQR)   Biologic therapy, n (%) | 31.6 (28.0 – 35.0)  1 (2.6)  23 (60.5)  15 (39.5)  0 (0.0)  8.4 (4.9 – 11.7)  11 (29.0) | 31.2 (28.4 – 33.6)  4 (6.5)  42 (67.7)  17 (27.4)  3 (4.8)  7.9 (4.5 – 11.7)  16 (25.8) | 0.200  0.395  0.463  0.210  0.169  0.637  0.731 |

**Supplementary Table 4.** Baseline differences between patients with metabolite measurements versus patients without metabolite measurements at each timepoint (continued)

| **Characteristic** | **Measurement present** | **No measurements** | **p-value** |
| --- | --- | --- | --- |
| Trimester 3-1^†^   - Age (y), median (IQR) - Active smoking, n (%) - IBD phenotype, n (%)   - CD - UC - IBD-U   - Disease duration (y), median (IQR) - Biologic therapy, n (%) | 31.0 (27.5 – 33.5)  1 (2.5)  25 (62.5)  12 (30.0)  3 (7.5)  6.9 (4.9 – 11.0)  8 (20.0) | 31.6 (28.8 – 34.5)  4 (6.7)  40 (66.7)  20 (33.3)  0 (0.0)  8.3 (4.6 – 12.2)  19 (31.7) | 0.247  0.349  0.669  0.726  **0.031**  0.385  0.198 |
| Trimester 3-2^†^   - Age (y), median (IQR) - Active smoking, n (%) - IBD phenotype, n (%)   - CD - UC - IBD-U   - Disease duration (y), median (IQR) - Biologic therapy, n (%) | 31.4 (27.4 – 33.7)  1 (3.7)  17 (63.0)  9 (33.3)  1 (3.7)  7.1 (4.4 – 12.6)  10 (37.0) | 31.3 (28.3 – 34.6)  4 (5.5)  48 (65.8)  23 (31.5)  2 (2.7)  8.2 (5.0 – 11.5)  17 (23.3) | 0.813  0.718  0.795  0.862  0.802  0.750  0.169 |
| Postpartum   - Age (y), median (IQR) - Active smoking, n (%) - IBD phenotype, n (%)   - CD - UC - IBD-U   - Disease duration (y), median (IQR) - Biologic therapy, n (%) | 31.4 (28.1 – 34.3)  1 (2.6)  27 (69.2)  11 (28.2)  1 (2.6)  8.3 (3.8 – 10.9)  9 (23.1) | 31.3 (28.1 – 34.1)  4 (6.6)  38 (62.3)  21 (34.4)  2 (3.3)  7.9 (5.0 – 12.2)  18 (29.5) | 0.651  0.371  0.478  0.515  0.838  0.543  0.480 |

Abbreviations: %, percentage; CD, Crohn’s disease; IBD, inflammatory bowel disease; IBD-U, inflammatory bowel disease unclassified; IQR, interquartile range; UC, ulcerative colitis; y, years;

† - The timepoints were defined as follows: trimester 1-1 (up to day 45), trimester 1-2 (day 46 – day 91), trimester 2-1 (day 92 – day 141), trimester 2-2 (day 142 – day 189), trimester 3-1 (day 190 – day 235), trimester 3-2 (day 236 – birth), postpartum (up to six months after birth).
